# Novel Insights into Dietary Phytosterol Utilization and Its Fate in Honey Bees (*Apis mellifera* L.)

**DOI:** 10.3390/molecules25030571

**Published:** 2020-01-28

**Authors:** Priyadarshini Chakrabarti, Hannah M. Lucas, Ramesh R. Sagili

**Affiliations:** Department of Horticulture, Oregon State University, 4017 Agriculture & Life Sciences Building, Corvallis, OR 97333, USA; hannah.lucas@oregonstate.edu (H.M.L.); ramesh.sagili@oregonstate.edu (R.R.S.)

**Keywords:** honey bee nutrition, 24-methylenecholesterol, phytosterol, insect physiology, endogenous sterol replacement, isotopomer

## Abstract

Poor nutrition is an important factor in global bee population declines. A significant gap in knowledge persists regarding the role of various nutrients (especially micronutrients) in honey bees. Sterols are essential micronutrients in insect diets and play a physiologically vital role as precursors of important molting hormones and building blocks of cellular membranes. Sterol requirements and metabolism in honey bees are poorly understood. Among all pollen sterols, 24-methylenecholesterol is considered the key phytosterol required by honey bees. Nurse bees assimilate this sterol from dietary sources and store it in their tissues as endogenous sterol, to be transferred to the growing larvae through brood food. This study examined the duration of replacement of such endogenous sterols in honey bees. The dietary ^13^C-labeled isotopomer of 24-methylenecholesterol added to artificial bee diet showed differential, progressive in vivo assimilation across various honey bee tissues. Significantly higher survival, diet consumption, head protein content and abdominal lipid content were observed in the dietary sterol-supplemented group than in the control group. These findings provide novel insights into phytosterol utilization and temporal pattern of endogenous 24-methylenecholesterol replacement in honey bees.

## 1. Introduction

Poor nutrition is one among the suite of factors cited as probable causes for honey bee colony declines over the past decade [1,2,3]. Research has shown that well-nourished honey bees survive longer, are better able to resist biotic and abiotic stressors and have enhanced immunity [4,5,6,7,8]. For all bee pollinators, the two principal dietary resources are pollen (their source of proteins, lipids phytochemicals and vitamins) and nectar (their primary source of carbohydrates and also vital phytochemicals) [4,5,9,10,11,12]. Pollen is additionally crucial because it is the only natural dietary source of important micronutrients for bees, for example: phytosterols [12]. Nurse bees consume pollen and are able to biosynthesize proteinaceous secretions from their hypopharyngeal glands. These proteinaceous secretions are progressively provisioned to the developing larvae [9,13,14].

Honey bees, like all insects, are sterol auxotrophs (unable to biosynthesize sterols) and are completely dependent on dietary resources for sterols. Nonetheless, these micronutrients are essential for various honey bee physiological functions. Sterols are precursors for insect molting hormones, act as signaling molecules for various developmental processes and are critical in maintaining the structural and functional integrity of the insect cellular membranes [15,16]. Therefore, a dietary source of sterols is paramount for optimal bee health. In honey bees, a specific phytosterol, 24-methylenecholesterol (24MC), resulted in the highest worker survival and the longest duration of brood production [17]. Other studies have documented large concentrations of 24MC in honey bee pupae [18,19] and reported that increased dietary intake of this sterol can increase honey bee survival and improve physiological markers such as head protein contents and abdominal lipid contents [20]. Thus, 24MC may be considered the most critical sterol in honey bees. While the role of dietary proteins [21], carbohydrates [22], minerals [23], omega-3-fatty acids [24], amino acids and vitamins [8] in bees have been relatively well studied, we currently lack an understanding of the requirements and metabolism of sterols. 

Previous research has shown that after consumption, honey bees store dietary sterols (primarily 24MC) in their tissues as endogenous sterols and then selectively transfer them from endogenous pools to the developing larvae through brood food [25,26]. That research provided important additions to our initial understanding of sterols in honey bees by conclusively showing movement of radiolabeled 24MC from experimental diets to adult honey bee tissues and then to the brood [26]. However, we still know very little about the timeline for this sterol utilization in honey bees.

Our study focused on gaining comprehensive insights on endogenous sterol regulation in honey bees. The primary objective of this study was to investigate the endogenous sterol replacement process in honey bees across various parts of the honey bee body (head, thorax and abdomen). In order to examine the temporal assimilation of the sterol molecule in honey bees, we quantified the amount of ^13^C stable carbon isotope in honey bee tissues. This isotope was supplemented via ^13^C-labeled 24MC in the synthetic diets (0.25% dry diet weight of ^13^C-labeled 24MC). Investigating the assimilation of the isotopomer within the bee tissues over time would provide further insights on the utilization of this particular sterol in honey bees.

Also, understanding the impacts of a labeled dietary constituent on bee physiology will be useful in designing long term nutritional studies using labeled diets. Hence, for both sterol-supplemented and control groups, we measured the consumption of the artificial diets and examined the physiological impacts of the labeled 24MC molecule on honey bees, by studying its effects on honey bee physiology (protein contents and abdominal lipid contents) and survival, as in a previous study [20]. Our findings provide novel insights into utilization and fate of sterols in honey bees. Information from this study can also be potentially used to understand the timeline for sterol supplementation and design a more complete artificial diet for honey bees, which is much needed given the widespread reports of declining bee health.

## 2. Results

### 2.1. Survival

**Figure 1:**
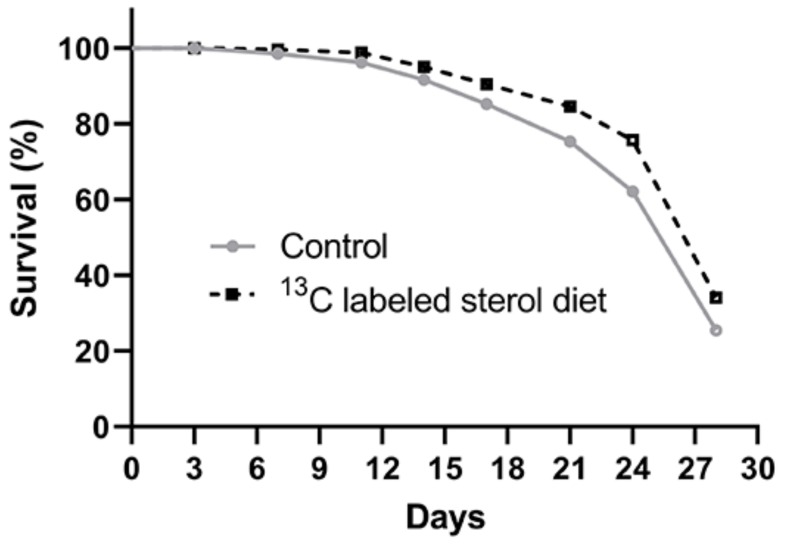
Kaplan-Meier survival curves for Control (honey bees fed diets with no sterol) and ^13^C-labeled sterol diet (honey bees fed diet containing 0.25% ^13^C-labeled 24-methylenecholesterol) groups.

Kaplan-Meier survival analyses revealed a significant difference in survival between the two experimental groups with honey bees in the ^13^C-24MC supplemented group (Log-rank Mantel-Cox test χ^2^ = 33.28, df = 1, *p* < 0.001; Figure 1) having higher survival. 

The survival proportions at the end of four weeks were 0.275 ± 0.02 and 0.354 ± 0.02 for the control group and sterol-supplemented group, respectively.

### 2.2. Consumption of Artificial Diets

**Figure 2:**
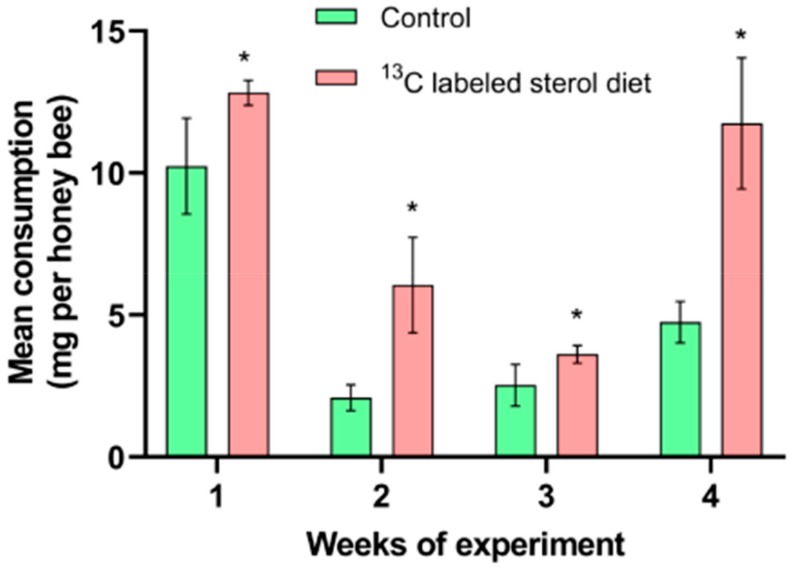
Mean consumption of artificial diets. Control: honey bees fed diet with no sterol; ^13^C-labeled sterol diet: honey bees fed diet containing 0.25% ^13^C-labeled 24-methylenecholesterol. Error bars indicate standard errors for means. * Indicates statistically significant differences at *p* < 0.05.

Honey bees in the sterol supplemented group consumed significantly more diet than bees in the control group during the experiment (week 1: t = −2.5664, df = 4, *p* < 0.05; week 2: t = −3.9423, df = 4, *p* < 0.05; week 3: t = −2.3844, df = 4, *p* < 0.05; week 4: t = −5.0106, df = 4, *p* < 0.05; Figure 2). The mean consumption per honey bee in the ^13^C-24MC supplemented group was 12.82 ± 0.25 mg, 6.05 ± 0.97 mg, 3.62 ± 0.18 mg and 11.74 ± 1.33 mg for weeks 1, 2, 3 and 4 respectively; and the consumption per honey bee for the control group was 10.24 ± 0.97 mg, 2.08 ± 0.26 mg, 2.52 ± 0.42 mg and 4.74 ± 0.42 mg for weeks 1, 2, 3 and 4 respectively.

### 2.3. Mean Head Protein and Abdominal Lipid Content in Honey Bees

**Figure 3:**
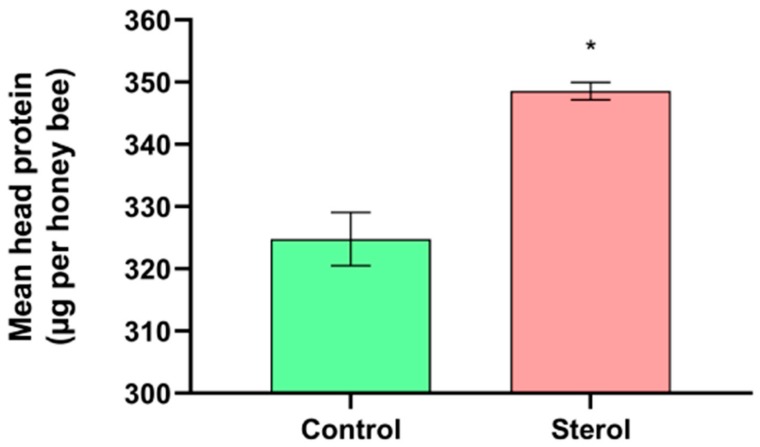
Mean head protein content (µg per bee) at the end of the four-week study. Control: bees fed diet with no sterol; Sterol: bees fed diet containing 0.25% ^13^C-labeled 24-methylenecholesterol. Error bars indicate standard errors for means. * Indicates statistically significant differences at *p* < 0.05.

At the end of the experiment, mean head protein content was significantly higher (t = −6.5328, df = 4, *p* < 0.05) in honey bees from the dietary ^13^C-24MC supplemented group (348.57 ± 1.41 µg) when compared to honey bees fed the control diet (324.79 ± 4.26 µg; Figure 3).

**Figure 4:**
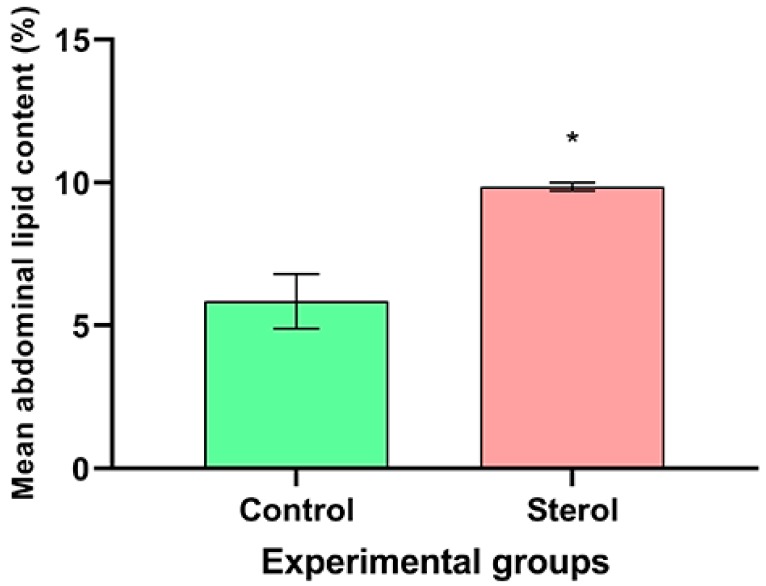
Mean abdominal lipid content (%) at the end of the four-week study. Control: honey bees fed diet with no sterol; Sterol: honey bees fed diet containing 0.25% ^13^C-labeled 24-methylenecholesterol. Error bars indicate standard errors for means. * Indicates statistically significant differences at *p* < 0.05.

Likewise, at the end of the experiment, dietary sterol supplemented bees had higher mean abdominal lipid content than bees in the control group (^13^C-24MC: 9.86 ± 0.08% dry abdominal weight; Control: 5.85 ± 0.55% dry abdominal weight; t = −7.1679, df = 4, *p* < 0.05; Figure 4).

### 2.4. Assimilation of ^13^C-Labeled 24MC across Honey Bee Tissues over Time

**Figure 5:**
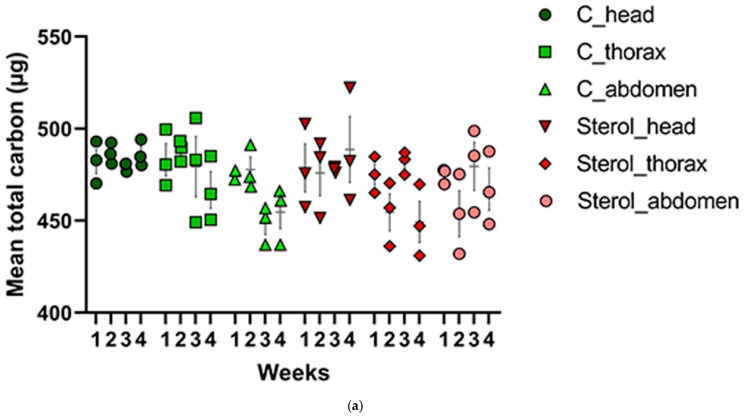

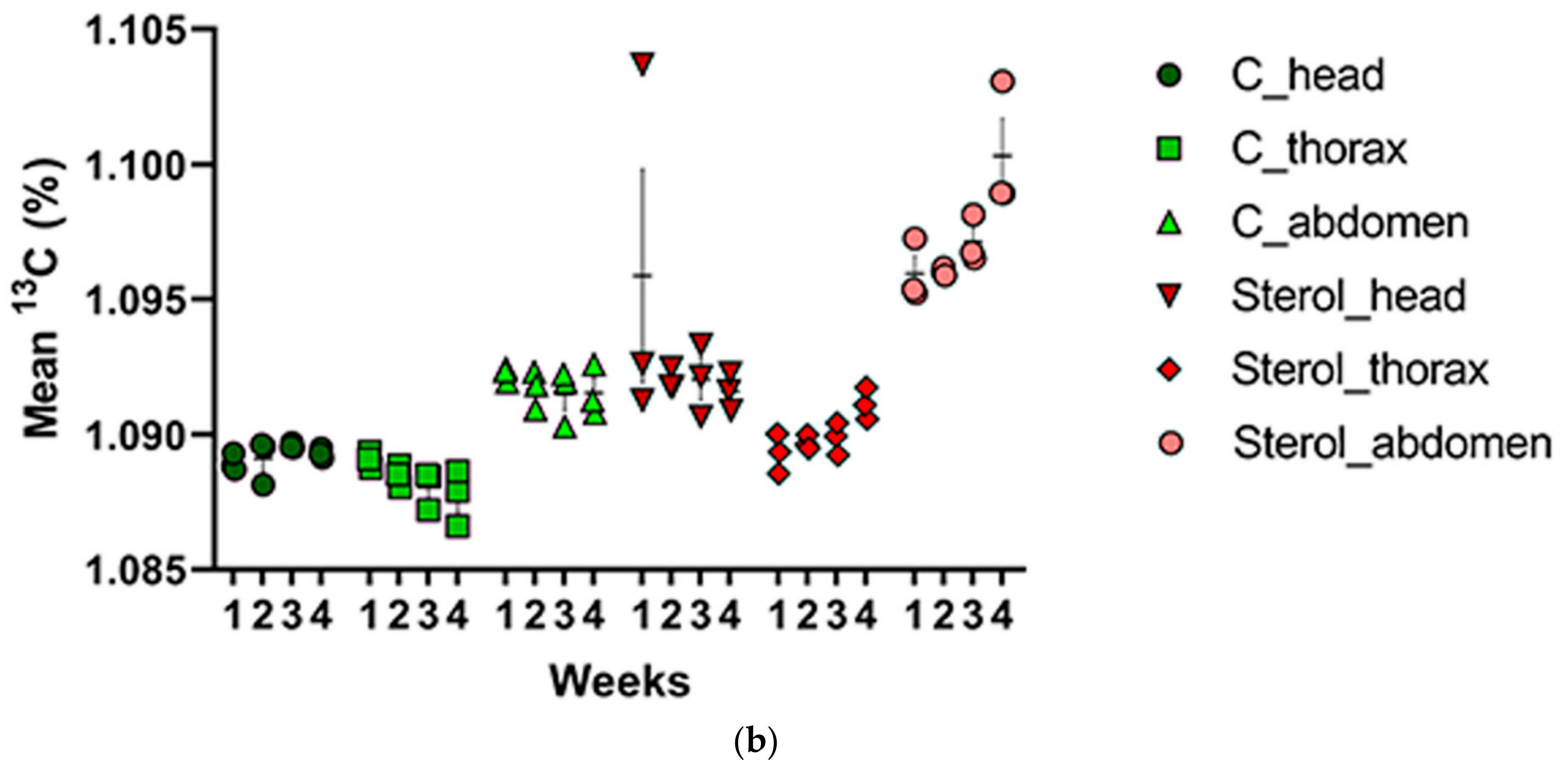
(**a**) Mean total carbon (μg) and (**b**) mean percentage of ^13^C for the control and treatment groups in all tissues across four weeks. C: honey bees fed diet with no sterol; Sterol: honey bees fed diet containing 0.25% ^13^C-labeled 24-methylenecholesterol. Bars indicate ± SEM.

All results from Tukey’s Post Hoc tests are provided in the supplementary information. Total carbon (µg) was analyzed in the heads, thoraces and abdomens of honey bees in both experimental groups (Table 1, Figure 5a) for every week. One-way ANOVA and Tukey Post Hoc Test (Table S1) indicated no significant difference between the tissues types (head, thorax and abdomen) across samples with respect to total carbon (µg) for weeks one (F_(5,12)_ = 0.2842, *p* = 0.9128), two (F_(5,12)_ = 2.935, *p* = 0.0587), three (F_(5,12)_ = 1.991, *p* = 0.1524) and four (F_(5,12)_ = 1.997, *p* = 0.1515) (Figure 5a).

Percentage of carbon-13 isotope was also analyzed for each week across tissue types for both control and sterol supplemented groups (Table 1, Figure 5b). No significant differences were observed during week 1 (F_(5,12)_ = 1.156, *p* = 0.3845) (Table S2). However, there were significant differences in percentage of ^13^C in tissues sampled across treatment groups at the end of two weeks (F_(5,12)_ = 86.19, *p* < 0.001), with abdominal tissues from the ^13^C-labeled 24MC groups exhibiting a significantly higher percentage of ^13^C compared to heads, which, in turn, had a significantly higher percentage of ^13^C than thoraces (Table S2). At the end of week three, a significant difference in percentage of ^13^C was also observed (F_(5,12)_ = 39.82, *p* < 0.001), with abdominal tissues from the sterol supplemented group exhibiting the highest percentages (Table S2). At the termination of the experiment (end of week four), abdominal tissues from ^13^C-labeled 24MC group still exhibited a significantly higher percentage of ^13^C compared to others (F_(5,12)_ = 40.50, *p* < 0.001) (Table S2).

When comparing the percentage of ^13^C in each tissue type for all four weeks in the control group, no significant differences were observed across weeks (Table S3). Thus, percentage of ^13^C contents were not significantly different (F_(3,8)_ = 1.042, *p* = 0.4251) in the heads of honey bees pertaining to control group across all four weeks. The same was true for thoracic (F_(3,8)_ = 2.203, *p* = 0.1654) and abdominal tissues (F_(3,8)_ = 0.5575, *p* = 0.6577). When percentages of ^13^C from the sterol supplemented group were analyzed, significant differences were observed for both thoracic (F_(3,8)_ = 5.705, *p* < 0.05) and abdominal tissues (F_(3, 8)_ = 6.417, *p* < 0.05) (Table S3). For thorax, percentages of ^13^C in week 4 were significantly higher than in week 1 (Supplementary Materials). For the abdominal tissues, percentages of ^13^C were significantly higher in week 4, when compared with week 1 and week 2 (Table S3). There were no significant differences (F_(3,8)_ = 0.9851, *p* = 0.4470) in the percentages of ^13^C in the sterol supplemented honey bee head tissues across all four weeks (Table S3). 

An overview of total carbon content and percentage ^13^C pertaining to ^13^C-labeled 24MC group is provided in Supplementary Figure S1. For the sterol-supplemented group, consumption of ^13^C, as well as ^13^C content (μg per bee) for each tissue type, at the end of each week are reported in Tables S4 and S5 (See Section 4 for calculations).

## 3. Discussion

To our knowledge, this is the first comprehensive study to investigate the temporal pattern of 24MC assimilation in honey bee tissues, and addresses some significant gaps in knowledge pertaining to honey bee sterol nutrition. We found that bees consumed higher amounts of the artificial diet that was supplemented with ^13^C-labeled 24MC; as seen in previous studies [17,20]. This suggests that honey bees were able to detect the presence of the sterol in the diets and the addition of the stable isotope did not affect any potentially phagostimulant effects of the 24MC molecule. In our study, survival (Figure 1), head protein content (Figure 3) and abdominal lipid content (Figure 4) were all significantly higher in bees that received diets containing ^13^C-labeled 24MC, when compared to bees in the control groups, even though carbohydrates and proteins were identical in the diets provided to both these groups. Previous studies have reported similar impacts of dietary sterols on various fitness traits in insects—for example increased longevity, locomotion and reproduction in adult ambrosia beetles [27]; significantly higher proportion of adult development in generalist grasshopper *Schistocerca americana* [28]; and increased head proteins, abdominal lipids and survival in caged honey bees in laboratory [20]. 

Hypopharyngeal glands and mandibular glands are the major brood food producing glands in honey bees [29]. One study [19] found that 24MC was in high quantities in the hypopharyngeal glands of nurse bees that were fed artificial diets supplemented with this sterol. Important secretory proteins, such as major royal jelly protein (MRJP1), are secreted by the nurse bee hypopharyngeal and mandibular glands [29,30] and a recent study [31] reported that MRJP1 oligomer can hold eight 24MC molecules. Further, with varying life cycle stages, age and nutritional needs, the amounts of lipids may also vary widely [32]. This variation is more relevant to honey bees, as newly emerged adult workers mature into foragers as they age [14,33,34]. A shift in the nutritional needs of honey bees thus accompanies their changing roles—from feeding larvae as nurses to collecting floral resources as foragers.

The physiological changes that occur in the brood food producing glands, and age polyethism in honey bees may explain the varying ^13^C isotope percentages observed across different honey bee tissues over the four weeks in this study. The primary goal of young adults (nurse bees) is provisioning larvae with sufficient proteinaceous secretions (brood food) [14], which are predominantly produced by the nurse bee hypopharyngeal glands [13]. Although the major components of brood food are water and protein [35], sterols are also present; 24MC being the most abundant among them [19,25]. This may explain why the newly emerged adults, caged and fed diets containing ^13^C-labeled 24MC, showed a higher accumulation of ^13^C in the heads (even though non-significant) during the beginning of the experiment (weeks 1 and 2) (Table 1).

As a nurse matures into a forager, she must store enough brood food resources in her body to potentially shift back to nursing during a colony emergency [13]. The abdominal fat body is the primary center for accumulation of lipids—sterols included. Thus, based on the information gleaned from this study and other studies [20], it appears that the bees supplemented with dietary sterols are able to replace the endogenous tissue sterols over time and store the excess sterols in the abdominal fat bodies. This may explain why we observed significantly higher abdominal fat contents (Figure 4) and percentages of ^13^C in abdomens toward the end of the experiment in the sterol-supplemented groups. Even though quantifying the labeled sterol molecule in honey bee tissues is beyond the scope of the present study, detection of ^13^C in our study is representative of the ^13^C labeled 24MC molecule. This is because previous research has shown that the major tissue sterol in honey bees is 24MC and honey bees are unable to convert C-24 alkyl phytosterols to cholesterol [19], hence retaining 24MC to transfer to the growing brood. 

The bees in the control group, which received diets without ^13^C-labeled 24MC, showed a significantly lower amount of ^13^C in the tissues. The bees for this experiment came from brood reared in colonies under natural field conditions, which were fed by nurse bees with access to some degree of pollen. The small quantity of ^13^C detected in control group samples could be from the modest amounts of pollen fed to larvae at later stages by the nurse bees. Plant tissues, including pollen grains, naturally contain stable isotopes [36,37]. Thus, newly emerged bees used in the study probably had endogenous sterols to varying degrees at the beginning of the study. However, thorough mixing of the all the newly emerged bees and then randomly allocating them to the experimental cages negated any potential effects of these endogenous sterols in newly emerged bees used in the study.

Addition of stable isotopes to artificial diets is useful in understanding the role of dietary constituents in an insect’s physiology, trophic ecology and nutritional enrichment [38,39]. In our study, stable carbon isotopes in the experimental diets demonstrated a complete endogenous sterol replacement between weeks three and four. Our study thus indicates the time span when colonies must have access to food containing this key phytosterol to sustain normal brood rearing, as inadequate food resources in a colony could induce cannibalism of young larvae [40]. The differential concentrations of this isotopomer across honey bee tissues also allude to its utilization by the bees over time. Although one previous study [19] has examined radiolabeled sterol localization in honey bees, the effects of labeled 24MC molecules on honey bee physiology and the timeline for complete endogenous sterol replacement were still poorly understood. Our findings thus address this gap in knowledge pertaining to endogenous sterol replacement dynamics.

Further, the data for diet consumption, head protein and abdominal lipid contents from the ^13^C-labeled 24MC supplemented group provide useful information regarding potential effects of stable isotope addition to insect artificial diets. The physiological responses to ^13^C-labeled 24MC diets observed in this study were similar to the responses observed in a previous study, where diets with unlabeled 24 MC were used [20]. This suggests that addition of carbon-13 stable isotope did not interfere with the structural integrity of the 24MC molecule and the ^13^C-labeled isotopomer of 24MC displays the same biochemical in vivo properties as the unlabeled compound, and thus can be used as a molecular tool in future studies on honey bee colony nutrition.

Overall, our study revealed novel insights pertaining to uptake of sterols and their role in honey bee nutritional physiology. Further, this study reports for the first time, the temporal pattern of endogenous 24MC replacement in honey bee tissues. Future research should look into the concentrations and impacts of 24-methylenecholesterol on entire honey bee colonies in a field realistic scenario. With multifactorial stressors contributing to pollinator decline, a fundamental knowledge of all nutritional needs is crucial to improve and sustain pollinator health. Insights gleaned from this study could help in formulation of a more complete diet for honey bees in the future.

## 4. Methods

The experiments performed are based on a previous study [18].

### 4.1. Collection of Bees and Formulation of Artificial Diets

A total of 18 frames, containing ready-to-emerge adult honey bees (*Apis mellifera*, L.), were collected from six sister-queen hives (three from each), that were maintained at the Oregon State University apiaries located in Corvallis (OR, USA). Sister queens ensured genetic similarities between the experimental honey bees. All the frames with ready-to-emerge bees were placed in an incubator at 33 °C, 55% RH (Percival Intellus I-36VL, Percival Scientific Inc., Perry, IA, USA) for the bees to emerge. After 24 h, all the bees that emerged were thoroughly mixed, and 100 newly emerged bees were randomly allocated to each experimental replicate cage for each treatment group. Each cylindrical cage used in the laboratory study was custom built with hardware cloth (1/4′′). An artificially formulated diet was placed on the cage floor. The bees were also given water and 40% sugar syrup (*w/v*) ad libitum from above. Evaporation control cages (empty cages with only sugar syrup, water and control diets) were also included to account for the loss due to evaporation. This study was conducted for four weeks. 

The artificial diet was formulated based on the diets used in previous studies [17,20] with few modifications to the sterol molecule (^13^C labeled 24MC). The sterol (labeled with ^13^C at the C-28 position, purchased from Expert Synthesis Solutions, London, ON, Canada) was dissolved in acetone, and then added to the artificial diet at a concentration of 0.25% dry diet weight. This sterol concentration was chosen, as it was the minimum concentration at which changes were observed in honey bee consumption and physiology in a previous study [20].

Each 2 g of the artificial diet that was formulated contained 810 mg of a complete amino acid powder containing all 20 amino acids (Nutricia, Zoetermeer, The Netherlands), 1.171 g sucrose (C&H Sugar, Crockett, CA, USA), 17 mg Wesson’s salt (MP Biochemicals, Irvine, CA, USA) and 2 mg zinc gluconate (Millipore Sigma, Burlington, MA, USA). The labeled sterol-acetone solution was mixed into the dry ingredients and acetone was allowed to evaporate off the diets under a fume hood for 24 h. Four μL of a B-vitamin mixture (Durvet, Blue Springs, MO, USA) and 450 µL of 40% sucrose syrup were then mixed into each diet to form a patty. In addition to the sterol treatment groups, we included a control group, which included the addition and evaporation of an equal volume of 100% acetone (no sterol). A patty thus formulated from two grams of dry diet weight was provided in each cage and all patties were replaced weekly. The two experimental groups were—^13^C labeled 24MC group (the labeled sterol added at 0.25% of dry diet weight) and a control group. There were three replicates per experimental group.

### 4.2. Survival Analyses

Bee mortality in each cage was recorded on days 3, 7, 11, 14, 17, 21, 24 and 28. Kaplan-Meier Survival analysis was performed based on previous studies [20,41,42]. The results are reported as χ^2^ values of Log-rank Mantel-Cox test, where 1 = event of interest and 0 = censored.

### 4.3. Diet Consumption

Diet consumption was recorded on a weekly basis and was calculated as the change in patty weight from the beginning of each week to the end of the week [20]. Evaporation control cages containing only artificial diets, sugar syrup and water were placed in the incubator as mentioned earlier. The change in diet weight within these empty cages represented the effects of evaporation. The mean weekly weight loss of diet patties in evaporation control cages was subtracted from the patty weight loss of each experimental cage and was adjusted to the average number of live honey bees in that experimental cage during the week.

### 4.4. Head Protein Content

The total head protein content of honey bees was estimated using a standard BCA assay (Pierce Biotech BCA Assay Kit, Thermo Scientific, Waltham, MA, USA) [20]. At the end of the experiment, the heads of ten bees were pooled together for each replicate cage. The pooled honey bee heads were homogenized in 600 µL of phosphate buffered saline (Sigma-Aldrich, St. Louis, MO, USA; pH 7.4) with one 3-mm tungsten carbide bead (Qiagen, Hilden, Germany) using a Tissue Lyser II (Qiagen; two rounds of 1.5 min at 30 oscillations s^−1^). Homogenized samples were then centrifuged at 4 °C for 6 min at 20,000× *g* (Eppendorf model 5430R, Eppendorf, Hamburg, Germany) to pellet the debris and the supernatant was collected. The microplate assay protocol in the kit instruction manual was followed and the absorbance was measured at 562 nm on a BioTek Synergy 2 plate reader (BioTek Instruments, Winooski, VT, USA).

### 4.5. Abdominal Lipid Contents

At the end of four weeks, for each experimental replicate, abdomens of ten honey bees were pooled and tested for abdominal fat content, following a protocol adapted from previous studies [20,43]. Each sample containing ten abdomens was dried for 72 h in a drying oven set at 45 °C (VWR, Radnor, PA, USA) and then weighed (Ohaus Pioneer Analytical, Parsippany, NJ, USA). The abdominal lipids were then solubilized by gently shaking each set of 10 abdomens in 3 mL anhydrous ethyl ether (Avantor Performance Materials Inc., Radnor Township, PA, USA) for 24 h on a microplate shaker (VWR). Next, the abdomens were dried under a fume hood for 72 h. The difference in weight between initial dry weight and final dry weight provided the abdominal lipid content. Results are reported as percent initial dry weight. The assay was performed once, at the end of the experiment.

### 4.6. Quantification of Carbon-13 (^13^C) in Honey Bees Tissues and Diets 

Five live honey bees were randomly collected from each experimental replicate (treatment and control groups) at the end of each week for the entire duration of the experiment. Honey bee bodies were divided into heads, thoraces and abdomens, and the guts were removed from abdomens to avoid ^13^C contamination from undigested food. The tissues were then dried in a drying oven (VWR) for 72 h at 45 °C and dry homogenized with one 3-mm tungsten carbide bead in a Tissue Lyser II (Qiagen; two rounds of 2 min at 30 oscillations sec^−1^). For a given week and tissue type, the five pulverized samples from each replicate were pooled together before analysis. Diets were similarly dried in a drying oven and homogenized. For each sample of dried tissue or diet, one milligram was weighed out on a Thermo Cahn Model 31 MicroBalance (Cahn Instruments Inc., Cerritos, CA, USA) in tin capsules (Elemental Microanalysis, Okehampton, UK). Quantification of total carbon and percentage of ^13^C were performed on a Sercon GSL preparation unit and a continuous flow PDZ-Europa 20/20 Isotope Ratio Mass Spectrometer (PDZ Europa Ltd., Northwich, UK) at the Stable Isotope Research Unit, Dept. of Crop and Soil Sciences, Oregon State University.

The ^13^C carbon excess values were obtained for individual tissue types (head, thorax and abdomen) using the following formula:

^13^C carbon excess = (% ^13^C in labeled-diet treated honey bee tissues) – (% ^13^C in control diet treated honey bee tissues) (1)

The ^13^C content (µg per bee) for individual tissue types (head, thorax and abdomen) was calculated as follows: (2)

^13^C content (µg per bee) = (^13^C carbon excess for tissue/100) × Total carbon (µg) (3)

The amount of ^13^C consumed by honey bees in the labeled-sterol treatment group was calculated as follows:

^13^C consumed (µg per bee) = Concentration × C_isotope_ × Diet_y_ × 1000 (4)

where Concentration = percentage of sterol in diet (0.25%); C_isotope_ = proportion of labeled carbons in 24MC molecule (1/28); Diet_y_ = consumption (mg per bee) in week “y”.

### 4.7. Statistical Analyses

All statistical analyses were performed using GraphPad Prism Version 7.03 software (GraphPad Software, San Diego, CA, USA). Data were checked for normality using Shapiro Wilk Test. Statistical significance was tested using two-tailed t-tests for two groups and One-Way ANOVA between multiple groups with Tukey’s Post Hoc test for multiple comparisons. Results are presented as mean values ± standard errors. Data was pooled for the replicates for each control and treatment group when comparing means.

## Figures and Tables

**Table 1:** Mean values ± SEM for % ^13^C and total carbon, across all bee tissue types over the four weeks of the experiment, where honey bees in the control group (C) were fed a control diet (no sterol); honey bees in the labeled 24-methylenecholesterol supplemented group (S) were fed a diet containing 0.25% ^13^C-labeled 24-methylenecholesterol.

Week	Tissue	S		C	
		^13^C Content (%)	Total Carbon (µg)	^13^C Content (%)	Total Carbon (µg)
1	Head	1.09587 ± 0.0039	478.67 ± 13.145	1.08895 ± 0.0002	482.116 ± 6.561
	Thorax	1.08931 ± 0.0004	475.06 ± 5.647	1.08907 ± 0.0001	483.189 ± 8.819
	Abdomen	1.09595 ± 0.0006	474.817 ± 2.455	1.09224 ± 0.0002	473.990 ± 1.645
2	Head	1.09202 ± 0.0002	475.967 ± 12.369	1.08911 ± 0.0004	486.616 ± 3.284
	Thorax	1.08971 ± 0.0001	454.56 ± 9.977	1.08848 ± 0.00023	488.414 ± 3.261
	Abdomen	1.09604 ± 0.0001	453.778 ± 12.494	1.09169 ± 0.0004	477.854 ± 6.811
3	Head	1.09204 ± 0.0008	477.893 ± 0.907	1.08958 ± 0.00006	479.495 ± 1.418
	Thorax	1.08987 ± 0.0003	481.817 ± 3.541	1.08805 ± 0.0004	479.347 ± 16.424
	Abdomen	1.09713 ± 0.0005	479.553 ± 13.115	1.09149 ± 0.0006	448.453 ± 5.970
4	Head	1.09159 ± 0.0004	488.66 ± 17.893	1.08931 ± 0.0001	486.447 ± 4.115
	Thorax	1.09114 ± 0.0003	449.36 ± 11.217	1.08772 ± 0.0005	466.739 ± 10.008
	Abdomen	1.10031 ± 0.0014	467.194 ± 11.433	1.09154 ± 0.0005	454.713 ± 8.995

## Supplementary Materials

The following are available online, Figure S1: Mean total carbon (primary vertical axis) and mean percentage of ^13^C (secondary vertical axis) found in honey bees fed a diet containing 0.25% ^13^C-labeled 24-methylenecholesterol. On the x-axis, h = head, t = thorax and a = abdomen. Numbers 1–4 (w1–w4) indicate weeks 1–4 of the experiments. Different alphabets indicate statistical significance at *p* < 0.05 for the % ^13^C datasets (blue lines on the grey bars), Table S1: Tukey’s Post Hoc results from One-way ANOVA tests for carbon contents across honey bee tissues for all experimental groups and weeks, Table S2: Tukey’s Post Hoc results from One-way ANOVA tests for ^13^C contents across honey bee tissues for all experimental groups and weeks, Table S3: Tukey’s Post Hoc results from One-way ANOVA tests for ^13^C contents in tissues across all four weeks for individual groups, Table S4: Consumption of ^13^C by labeled 24-methylenecholesterol supplemented honey bees (μg per bee), Table S5: ^13^C contents of the three tissue types from the sterol-supplemented group are provided across four weeks.
