# Supplementary material for: Novel Insights into Dietary Phytosterol Utilization and Its Fate in Honey Bees (Apis mellifera L.)
Source: Molecules. 2020 Jan 28;25(3):571. doi: 10.3390/molecules25030571 (PMC7036750; doi:10.3390/molecules25030571)
Supplement: Supplementary file 1 [file molecules-25-00571-s001.pdf]

Supplementary figure 1: Mean total carbon (primary vertical axis) and mean percentage of 13C (secondary vertical axis) found in honey bees fed a diet containing 0.25% 13C-labeled 24-methylenecholesterol. On the x-axis, h = head, t = thorax and a = abdomen. Numbers 1 – 4 (w1-w4) indicate weeks 1 – 4 of the experiments. Different alphabets indicate statistical significance at $p < 0.05$ for the % ^{13}C datasets (blue lines on the grey bars).

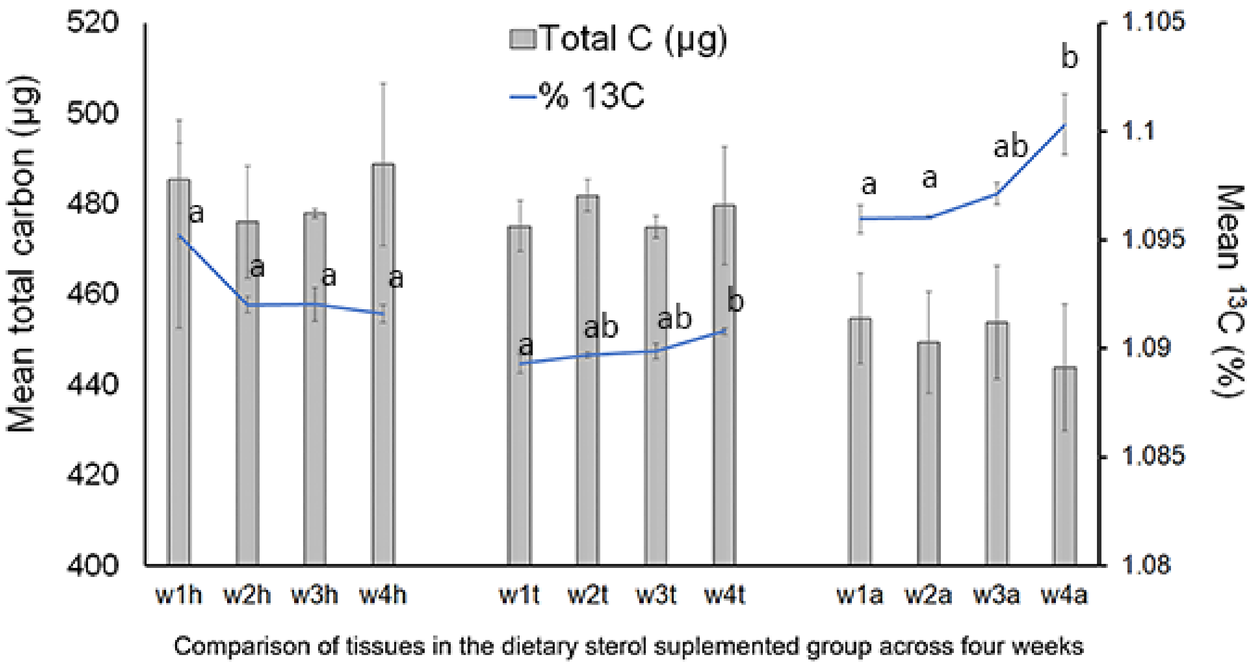

Supplementary table 1: Tukey's Post Hoc results from One-way ANOVA tests for carbon contents across honey bee tissues for all experimental groups and weeks.

(A) Week1

**Tukey's multiple comparisons test**	**Mean Diff.**	**95.00% CI of diff.**	**Significant?**	**Summary**	**Adjusted P Value**
C_head vs. C_thorax	-1.073	-36.53 to 34.38	No	ns	>0.9999
C_head vs. C_abdomen	8.126	-27.33 to 43.58	No	ns	0.9677
C_head vs. 13C_head	3.446	-32.01 to 38.90	No	ns	0.9994
C_head vs. 13C_thorax	7.056	-28.40 to	No	ns	0.9823

		42.51			
C_head vs. 13C_abdomen	7.299	-28.16 to 42.76	No	ns	0.9795
C_thorax vs. C_abdomen	9.199	-26.26 to 44.65	No	ns	0.9465
C_thorax vs. 13C_head	4.519	-30.94 to 39.98	No	ns	0.9977
C_thorax vs. 13C_thorax	8.129	-27.33 to 43.59	No	ns	0.9676
C_thorax vs. 13C_abdomen	8.372	-27.08 to 43.83	No	ns	0.9634
C_abdomen vs. 13C_head	-4.68	-40.14 to 30.78	No	ns	0.9973
C_abdomen vs. 13C_thorax	-1.07	-36.53 to 34.39	No	ns	>0.9999
C_abdomen vs. 13C_abdomen	-0.8264	-36.28 to 34.63	No	ns	>0.9999
13C_head vs. 13C_thorax	3.61	-31.85 to 39.07	No	ns	0.9992
13C_head vs. 13C_abdomen	3.853	-31.60 to 39.31	No	ns	0.9989
13C_thorax vs. 13C_abdomen	0.2433	-35.21 to 35.70	No	ns	>0.9999

(B) Week2

**Tukey's multiple comparisons test**	**Mean Diff.**	**95.00% CI of diff.**	**Significant?**	**Summary**	**Adjusted P Value**
C_head vs. C_thorax	-1.798	-44.12 to 40.52	No	ns	>0.9999
C_head vs. C_abdomen	8.762	-33.56 to 51.08	No	ns	0.979
C_head vs. 13C_head	10.65	-31.67 to 52.97	No	ns	0.9526
C_head vs. 13C_thorax	32.06	-10.27 to 74.38	No	ns	0.1857
C_head vs. 13C_abdomen	32.84	-9.482 to 75.16	No	ns	0.169
C_thorax vs. C_abdomen	10.56	-31.76 to 52.88	No	ns	0.9542
C_thorax vs. 13C_head	12.45	-29.87 to 54.77	No	ns	0.9133
C_thorax vs. 13C_thorax	33.85	-8.467 to 76.18	No	ns	0.1492

C_thorax vs. 13C_abdomen	34.64	-7.684 to 76.96	No	ns	0.1354
C_abdomen vs. 13C_head	1.888	-40.43 to 44.21	No	ns	>0.9999
C_abdomen vs. 13C_thorax	23.29	-19.03 to 65.62	No	ns	0.4735
C_abdomen vs. 13C_abdomen	24.08	-18.24 to 66.40	No	ns	0.4406
13C_head vs. 13C_thorax	21.41	-20.91 to 63.73	No	ns	0.5567
13C_head vs. 13C_abdomen	22.19	-20.13 to 64.51	No	ns	0.5217
13C_thorax vs. 13C_abdomen	0.7833	-41.54 to 43.10	No	ns	>0.9999

(C) Week3

**Tukey's multiple comparisons test**	**Mean Diff.**	**95.00% CI of diff.**	**Significant?**	**Summary**	**Adjusted P Value**
C_head vs. C_thorax	0.1478	-42.90 to 43.20	No	ns	>0.9999
C_head vs. C_abdomen	31.04	-12.01 to 74.09	No	ns	0.2227
C_head vs. 13C_head	1.602	-41.45 to 44.65	No	ns	>0.9999
C_head vs. 13C_thorax	-2.322	-45.37 to 40.73	No	ns	>0.9999
C_head vs. 13C_abdomen	-0.05831	-43.11 to 42.99	No	ns	>0.9999
C_thorax vs. C_abdomen	30.89	-12.15 to 73.94	No	ns	0.2265
C_thorax vs. 13C_head	1.454	-41.59 to 44.50	No	ns	>0.9999
C_thorax vs. 13C_thorax	-2.469	-45.52 to 40.58	No	ns	>0.9999
C_thorax vs. 13C_abdomen	-0.2061	-43.25 to 42.84	No	ns	>0.9999
C_abdomen vs. 13C_head	-29.44	-72.49 to 13.61	No	ns	0.2665
C_abdomen vs. 13C_thorax	-33.36	-76.41 to 9.684	No	ns	0.1698
C_abdomen vs. 13C_abdomen	-31.1	-74.15 to 11.95	No	ns	0.2212
13C_head vs.	-3.923	-46.97 to	No	ns	0.9995

13C_thorax		39.12			
13C_head vs. 13C_abdomen	-1.66	-44.71 to 41.39	No	ns	>0.9999
13C_thorax vs. 13C_abdomen	2.263	-40.78 to 45.31	No	ns	>0.9999

(D) Week4

Tukey's multiple comparisons test	Mean Diff.	95.00% CI of diff.	Significant?	Summary	Adjusted P Value
C_head vs. C_thorax	19.71	-34.27 to 73.68	No	ns	0.8164
C_head vs. C_abdomen	31.73	-22.24 to 85.71	No	ns	0.408
C_head vs. 13C_head	-2.213	-56.19 to 51.76	No	ns	>0.9999
C_head vs. 13C_thorax	37.09	-16.89 to 91.06	No	ns	0.2625
C_head vs. 13C_abdomen	19.25	-34.72 to 73.23	No	ns	0.8297
C_thorax vs. C_abdomen	12.03	-41.95 to 66.00	No	ns	0.9713
C_thorax vs. 13C_head	-21.92	-75.90 to 32.05	No	ns	0.7462
C_thorax vs. 13C_thorax	17.38	-36.60 to 71.35	No	ns	0.8796
C_thorax vs. 13C_abdomen	-0.4545	-54.43 to 53.52	No	ns	>0.9999
C_abdomen vs. 13C_head	-33.95	-87.92 to 20.03	No	ns	0.3426
C_abdomen vs. 13C_thorax	5.353	-48.62 to 59.33	No	ns	0.9993
C_abdomen vs. 13C_abdomen	-12.48	-66.46 to 41.50	No	ns	0.9665
13C_head vs. 13C_thorax	39.3	-14.68 to 93.28	No	ns	0.2151
13C_head vs. 13C_abdomen	21.47	-32.51 to 75.44	No	ns	0.7612
13C_thorax vs. 13C_abdomen	-17.83	-71.81 to 36.14	No	ns	0.8683

Supplementary table 2: Tukey's Post Hoc results from One-way ANOVA tests for ^{13}C contents across honey bee tissues for all experimental groups and weeks.

(A) Week1

**Tukey's multiple comparisons test**	**Mean Diff.**	**95.00% CI of diff.**	**Significant?**	**Summary**	**Adjusted P Value**
C_head vs. C_thorax	-0.00012	-0.007924 to 0.007690	No	ns	>0.9999
C_head vs. C_abdomen	-0.00329	-0.01109 to 0.004521	No	ns	0.7192
C_head vs. 13C_head	-0.00692	-0.01473 to 0.0008889	No	ns	0.0941
C_head vs. 13C_thorax	-0.00036	-0.008169 to 0.007445	No	ns	>0.9999
C_head vs. 13C_abdomen	-0.007	-0.01481 to 0.0008074	No	ns	0.0889
C_thorax vs. C_abdomen	-0.00317	-0.01098 to 0.004638	No	ns	0.7467
C_thorax vs. 13C_head	-0.0068	-0.01461 to 0.001006	No	ns	0.1021
C_thorax vs. 13C_thorax	-0.00024	-0.008052 to 0.007562	No	ns	>0.9999
C_thorax vs. 13C_abdomen	-0.00688	-0.01469 to 0.0009248	No	ns	0.0965
C_abdomen vs. 13C_head	-0.00363	-0.01144 to 0.004175	No	ns	0.6349
C_abdomen vs. 13C_thorax	0.002924	-0.004883 to 0.01073	No	ns	0.8011
C_abdomen vs. 13C_abdomen	-0.00371	-0.01152 to 0.004093	No	ns	0.6147
13C_head vs. 13C_thorax	0.006556	-0.001251 to 0.01436	No	ns	0.1209
13C_head vs. 13C_abdomen	-8.2E-05	-0.007888 to 0.007725	No	ns	>0.9999
13C_thorax vs. 13C_abdomen	-0.00664	-0.01444 to 0.001169	No	ns	0.1143

(B) Week2

Tukey's multiple comparisons test	Mean Diff.	95.00% CI of diff.	Significant?	Summary	Adjusted P Value
C_head vs. C_thorax	0.00063	-0.0007868 to 0.002046	No	ns	0.6747
C_head vs. C_abdomen	-0.00258	-0.004001 to -0.001168	Yes	***	0.0006
C_head vs. 13C_head	-0.00291	-0.004328 to -0.001495	Yes	***	0.0002
C_head vs. 13C_thorax	-0.0006	-0.002019 to 0.0008137	No	ns	0.7106
C_head vs. 13C_abdomen	-0.00693	-0.008346 to -0.005513	Yes	****	<0.0001
C_thorax vs. C_abdomen	-0.00321	-0.004630 to -0.001798	Yes	****	<0.0001
C_thorax vs. 13C_head	-0.00354	-0.004957 to -0.002124	Yes	****	<0.0001
C_thorax vs. 13C_thorax	-0.00123	-0.002649 to 0.0001841	No	ns	0.1027
C_thorax vs. 13C_abdomen	-0.00756	-0.008976 to -0.006143	Yes	****	<0.0001
C_abdomen vs. 13C_head	-0.00033	-0.001743 to 0.001090	No	ns	0.9667
C_abdomen vs. 13C_thorax	0.001982	0.0005652 to 0.003398	Yes	**	0.0053
C_abdomen vs. 13C_abdomen	-0.00435	-0.005762 to -0.002929	Yes	****	<0.0001
13C_head vs. 13C_thorax	0.002309	0.0008922 to 0.003725	Yes	**	0.0015
13C_head vs. 13C_abdomen	-0.00402	-0.005435 to -0.002602	Yes	****	<0.0001
13C_thorax vs. 13C_abdomen	-0.00633	-0.007744 to -0.004911	Yes	****	<0.0001

(C) Week3

Tukey's multiple comparisons test	Mean Diff.	95.00% CI of diff.	Significant?	Summary	Adjusted P Value
C_head vs. C_thorax	0.001524	-0.0008622 to 0.003909	No	ns	0.3282
C_head vs. C_abdomen	-0.00191	-0.004298 to 0.0004736	No	ns	0.148
C_head vs. 13C_head	-0.00246	-0.004850 to -7.798e-005	Yes	*	0.0416
C_head vs. 13C_thorax	-0.0003	-0.002683 to 0.002088	No	ns	0.9979
C_head vs. 13C_abdomen	-0.00756	-0.009948 to -0.005176	Yes	****	<0.0001
C_thorax vs. C_abdomen	-0.00344	-0.005822 to -0.001050	Yes	**	0.0042
C_thorax vs. 13C_head	-0.00399	-0.006373 to -0.001602	Yes	**	0.0012
C_thorax vs. 13C_thorax	-0.00182	-0.004207 to 0.0005648	No	ns	0.1803
C_thorax vs. 13C_abdomen	-0.00909	-0.01147 to -0.006700	Yes	****	<0.0001
C_abdomen vs. 13C_head	-0.00055	-0.002937 to 0.001834	No	ns	0.9665
C_abdomen vs. 13C_thorax	0.001615	-0.0007710 to 0.004001	No	ns	0.2754
C_abdomen vs. 13C_abdomen	-0.00565	-0.008035 to -0.003264	Yes	****	<0.0001
13C_head vs. 13C_thorax	0.002166	-0.0002194 to 0.004552	No	ns	0.0835
13C_head vs. 13C_abdomen	-0.0051	-0.007484 to -0.002712	Yes	***	0.0001
13C_thorax vs. 13C_abdomen	-0.00727	-0.009650 to -0.004879	Yes	****	<0.0001

(D) Week4

Tukey's multiple comparisons test	Mean Diff.	95.00% CI of diff.	Significant?	Summary	Adjusted P Value
C_head vs. C_thorax	0.001592	-0.001674 to 0.004858	No	ns	0.592
C_head vs. C_abdomen	-0.00223	-0.005498 to 0.001035	No	ns	0.2673
C_head vs. 13C_head	-0.00228	-0.005544 to 0.0009887	No	ns	0.25
C_head vs. 13C_thorax	-0.00183	-0.005092 to 0.001440	No	ns	0.4578
C_head vs. 13C_abdomen	-0.011	-0.01427 to -0.007734	Yes	****	<0.0001
C_thorax vs. C_abdomen	-0.00382	-0.007090 to -0.0005575	Yes	*	0.019
C_thorax vs. 13C_head	-0.00387	-0.007136 to -0.0006033	Yes	*	0.0176
C_thorax vs. 13C_thorax	-0.00342	-0.006684 to -0.0001522	Yes	*	0.0384
C_thorax vs. 13C_abdomen	-0.01259	-0.01586 to -0.009326	Yes	****	<0.0001
C_abdomen vs. 13C_head	-4.6E-05	-0.003312 to 0.003220	No	ns	>0.9999
C_abdomen vs. 13C_thorax	0.000405	-0.002861 to 0.003671	No	ns	0.998
C_abdomen vs. 13C_abdomen	-0.00877	-0.01203 to -0.005502	Yes	****	<0.0001
13C_head vs. 13C_thorax	0.000451	-0.002815 to 0.003717	No	ns	0.9966
13C_head vs. 13C_abdomen	-0.00872	-0.01199 to -0.005457	Yes	****	<0.0001
13C_thorax vs. 13C_abdomen	-0.00917	-0.01244 to -0.005908	Yes	****	<0.0001

Supplementary table 3: Tukey's Post Hoc results from One-way ANOVA tests for ^{13}C contents in tissues across all four weeks for individual groups.

Groups	Tukey's multiple comparisons test	Mean Diff.	95.00% CI of diff.	Significant?	Summary	Adjusted P Value
Control head	w1h vs. w2h	-0.00015	-0.001351 to 0.001043	No	ns	0.9747
Control head	w1h vs. w3h	-0.00062	-0.001820 to 0.0005741	No	ns	0.3984
Control head	w1h vs. w4h	-0.00036	-0.001558 to 0.0008360	No	ns	0.7718
Control head	w2h vs. w3h	-0.00047	-0.001666 to 0.0007284	No	ns	0.6135
Control head	w2h vs. w4h	-0.00021	-0.001404 to 0.0009902	No	ns	0.9431
Control head	w3h vs. w4h	0.000262	-0.0009351 to 0.001459	No	ns	0.8941
Control thorax	w1t vs. w2t	0.000593	-0.001184 to 0.002369	No	ns	0.7167
Control thorax	w1t vs. w3t	0.001018	-0.0007582 to 0.002794	No	ns	0.3252
Control thorax	w1t vs. w4t	0.001348	-0.0004279 to 0.003125	No	ns	0.1479
Control thorax	w2t vs. w3t	0.000425	-0.001351 to 0.002202	No	ns	0.8672
Control thorax	w2t vs. w4t	0.000756	-0.001021 to 0.002532	No	ns	0.5534
Control thorax	w3t vs. w4t	0.00033	-0.001446 to 0.002107	No	ns	0.9306
Control abdomen	w1a vs. w2a	0.000547	-0.001532 to 0.002626	No	ns	0.8329
Control abdomen	w1a vs. w3a	0.000751	-0.001328 to 0.002830	No	ns	0.6681
Control abdomen	w1a vs. w4a	0.000693	-0.001386 to 0.002772	No	ns	0.7171
Control abdomen	w2a vs. w3a	0.000203	-0.001875 to 0.002282	No	ns	0.9885
Control abdomen	w2a vs. w4a	0.000146	-0.001933 to 0.002225	No	ns	0.9957
Control abdomen	w3a vs. w4a	-5.7E-05	-0.002136 to 0.002021	No	ns	0.9997
Sterol head	w1h vs. w2h	0.003852	-0.005294 to 0.01300	No	ns	0.5608
Sterol head	w1h vs. w3h	0.003831	-0.005315 to 0.01298	No	ns	0.5649

Sterol head	w1h vs. w4h	0.00428	-0.004867 to 0.01343	No	ns	0.4809
Sterol head	w2h vs. w3h	-2.1E-05	-0.009168 to 0.009126	No	ns	>0.9999
Sterol head	w2h vs. w4h	0.000427	-0.008720 to 0.009574	No	ns	0.9987
Sterol head	w3h vs. w4h	0.000448	-0.008699 to 0.009595	No	ns	0.9985
Sterol thorax	w1t vs. w2t	-0.00039	-0.001892 to 0.001102	No	ns	0.832
Sterol thorax	w1t vs. w3t	-0.00056	-0.002055 to 0.0009384	No	ns	0.6467
Sterol thorax	w1t vs. w4t	-0.00183	-0.003322 to -0.0003286	Yes	*	0.0189
Sterol thorax	w2t vs. w3t	-0.00016	-0.001660 to 0.001333	No	ns	0.9843
Sterol thorax	w2t vs. w4t	-0.00143	-0.002927 to 6.638e-005	No	ns	0.0611
Sterol thorax	w3t vs. w4t	-0.00127	-0.002764 to 0.0002297	No	ns	0.1
Sterol abdomen	w1a vs. w2a	-8.5E-05	-0.003735 to 0.003566	No	ns	0.9998
Sterol abdomen	w1a vs. w3a	-0.00119	-0.004836 to 0.002465	No	ns	0.7325
Sterol abdomen	w1a vs. w4a	-0.00436	-0.008012 to -0.0007111	Yes	*	0.0211
Sterol abdomen	w2a vs. w3a	-0.0011	-0.004751 to 0.002550	No	ns	0.7719
Sterol abdomen	w2a vs. w4a	-0.00428	-0.007928 to -0.0006266	Yes	*	0.0233
Sterol abdomen	w3a vs. w4a	-0.00318	-0.006827 to 0.0004742	No	ns	0.0899

Supplementary table 4. Consumption of ^{13}C by labeled 24-methylenecholesterol supplemented honey bees (µg per bee).

Weeks.	***Consumption of ^{13}C (µg per bee)***
**1**	1.1447 ± 0.0223
**2**	0.540 ± 0.0867
**3**	0.323 ± 0.0161
**4**	1.0487 ± 0.119

Supplementary table 5. ^{13}C contents of the three tissue types from the sterol-supplemented group are provided across four weeks. The values provided are mean values ± SE.

Weeks	***^{13}C contents (µg per tissue types)***		
	Head	***Thorax***	***Abdomen***
**1**	0.0358 ± 0.020	0.0121 ±0.0019	0.0361 ± 0.0032
**2**	0.01324 ± 0.001	0.0131 ± 0.0006	0.03148 ± 0.0006
**3**	0.01325 ± 0.004	0.0102 ± 0.0017	0.03381 ± 0.0016
**4**	0.0199 ± 0.003	0.0209 ± 0.0013	0.05213 ± 0.0067
